# Ascorbate Peroxidase Neofunctionalization at the Origin of APX-R and APX-L: Evidence from Basal Archaeplastida

**DOI:** 10.3390/antiox10040597

**Published:** 2021-04-13

**Authors:** Fernanda Lazzarotto, Paloma Koprovski Menguer, Luiz-Eduardo Del-Bem, Marcel Zámocký, Márcia Margis-Pinheiro

**Affiliations:** 1 Centro de Biotecnologia, Programa de Pós-Graduação em Biologia Celular e Molecular, Universidade Federal do Rio Grande do Sul, Porto Alegre 91509-900, Brazil; fernanda.lazzarotto@ufrgs.br (F.L.); paloma.menguer@ufrgs.br (P.K.M.); 2 Departamento de Botânica, Instituto de Ciências Biológicas, Universidade Federal De Minas Gerais, Belo Horizonte 31270-901, Brazil; delbem@ufmg.br; 3 Laboratory of Phylogenomic Ecology, Institute of Molecular Biology, Slovak Academy of Sciences, SK-84551 Bratislava, Slovakia; marcel.zamocky@savba.sk; 4 Programa de Pós-Graduação em Genética e Biologia Molecular, Departamento de Genética, Universidade Federal do Rio Grande do Sul, Porto Alegre 91509-900, Brazil

**Keywords:** ascorbate peroxidase—APX, APX-R, APX-L, catalytic sites, substrate, protein divergence

## Abstract

Ascorbate peroxidases (APX) are class I members of the Peroxidase-Catalase superfamily, a large group of evolutionarily related but rather divergent enzymes. Through mining in public databases, unusual subsets of APX homologs were identified, disclosing the existence of two yet uncharacterized families of peroxidases named ascorbate peroxidase-related (APX-R) and ascorbate peroxidase-like (APX-L). As APX, APX-R harbor all catalytic residues required for peroxidatic activity. Nevertheless, proteins of this family do not contain residues known to be critical for ascorbate binding and therefore cannot use it as an electron donor. On the other hand, APX-L proteins not only lack ascorbate-binding residues, but also every other residue known to be essential for peroxidase activity. Through a molecular phylogenetic analysis performed with sequences derived from basal Archaeplastida, the present study discloses the existence of hybrid proteins, which combine features of these three families. The results here presented show that the prevalence of hybrid proteins varies among distinct groups of organisms, accounting for up to 33% of total APX homologs in species of green algae. The analysis of this heterogeneous group of proteins sheds light on the origin of APX-R and APX-L and suggests the occurrence of a process characterized by the progressive deterioration of ascorbate-binding and catalytic sites towards neofunctionalization.

## 1. Introduction

Ascorbate peroxidases (APX) (EC 1.11.1.11) are heme b enzymes that catalyze the reduction of hydrogen peroxide (H_2_O_2_) into water in oxygenic photosynthetic organisms, in a process dependent on ascorbate [1]. Based on its peculiar chemical structure, ascorbate is considered to be a successful antioxidant from an evolutionary perspective, acting together with glutathione to ensure efficient detoxification of hydrogen peroxide in photosynthetic organisms, and further allowing the establishment and rise of the APX family [2]. APX are usually codified by small gene families, leading to the occurrence of distinguished isoforms targeted to the cytosol, plastids, mitochondrion, and peroxisomes, which differ not only on subcellular localization, but also in substrate affinity, dimer formation, and in the presence of transmembrane domains [1,3,4,5].

As for other heme-containing enzymes, the catalytic mechanism of ascorbate peroxidases involves the formation of an iron-oxidized reactive intermediate known as compound I and its subsequent reduction by the substrate, in this particular case ascorbate, in two sequential electron transfer steps [6,7]. The catalytic mechanism, crystal structure, and ascorbate binding of APX were extensively studied in the past 30 years [8,9,10,11,12,13,14]. Through crystallographic information and site-directed mutagenesis, it was possible to dissect the main residues implicated in APX activity, which are allocated in two structural domains surrounding the heme [15]. The C-terminal domain contains the proximal histidine (His163—numbered following *Pisum sativum* APx1 sequence), which is hydrogen-bonded to the heme moiety. The N-terminal domain harbors the distal histidine (His42) and an arginine (Arg38) that are crucial for the heterolytic cleavage of H_2_O_2_, as well as a tryptophan residue (Trp41) implicated in heme binding and coordination [15,16,17,18,19]. The interaction of APX with ascorbate is dependent on an arginine located nearby the proximal histidine (Arg172), since mutations in this residue were sufficient to abolish APX activity towards this substrate [11,12,13,20]. Nevertheless, N-terminal lysine (Lys30) and cysteine (Cys32) also seem to contribute to ascorbate binding, but to a much lesser extent [11,12].

The APX family is part of the peroxidase-catalase superfamily (previously known as non-animal peroxidase superfamily), a group of evolutionarily related enzymes distributed among bacteria, archaea, algae, fungi, plants, cnidaria, and even ecdysozoa [21,22]. Even though APX are mostly found in photosynthetic organisms, these enzymes were also purified from protozoans and even mammals, indicating a broader prevalence for the APX family which remains to be further analyzed and explored [23,24]. Despite the rather low sequence homology among superfamily members, proteins that belong to this group share common features like folding, typical secondary structure, and catalytic domains, most likely as a consequence of their common origin. The most accepted theory to explain the superfamily establishment is based on countless rounds of gene duplication and diversification from an ancestral prokaryotic peroxidase and is tightly associated with the endosymbiotic events that led to mitochondria and chloroplast acquisition [25]. Molecular phylogeny analyses separate the superfamily members into three well-supported classes, among which over ten families are accommodated [25,26,27]. Previously, through mining in public databases, an unusual subset of APX proteins that did not cluster with classic APX members in phylogenetic analyses were identified. These proteins show several conserved substitutions when compared to classical APX, which include the substitution of Trp41 by phenylalanine, a change that was reported for other superfamily peroxidases (which integrate Classes II and III), and, more importantly, the lack of Arg172, suggesting they should not be able to use ascorbate as a catalytic substrate. After a refined phylogenetic analysis, the separation of this group of APX homologs into a distinct family of heme peroxidases could be further confirmed, leading to the description of a new family, which was named ascorbate peroxidase-related (APX-R) [28]. Members of this family are mostly predicted to contain a transit peptide to chloroplasts and/or mitochondria import, and to be encoded by a single-copy gene in most plant genomes. Genomic analyses performed in land plants showed that genes that encode for APX-R return to single-copy arrangements even after repeated intra-chromosomic duplications involving these loci, suggesting the occurrence of gene dosage effect related to members of this family. Genes that encode for APX-R also display conserved structure (regarding number and size of exons), which differs significantly from the one described for APX [28]. Functional analysis demonstrated that APX-R knockdown disturbed the redox metabolism in rice (*Oryza sativa*), while analyses conducted in Arabidopsis knockout plants revealed the importance of this peroxidase to oxidative protection and hormonal steadiness in seeds, as well as unveiled the existence of a post-translational mechanism to regulate APX-R accumulation during photomorphogenesis [28,29,30]. The enzymatic activity of APX-R was also investigated recently, and in vitro experiments have now confirmed that APX-R is a functional heme peroxidase which does not rely on ascorbate to reduce H_2_O_2_ [30]. Nevertheless, the natural substrate of APX-R remains to be determined.

After a comprehensive phylogenetic analysis, APX-R could be further classified as a class I member of the peroxidase-catalase superfamily, along with APX, catalase-peroxidase (KATG), cytochrome-c peroxidase (CCP), and APX-CCP families [27]. Moreover, this analysis also revealed the existence of another well-supported family composed of a small subset of proteins currently annotated as APX homologs, which was named ascorbate peroxidase-like (APX-L). In contrast to what was observed for APX-R, APX-L proteins do not harbor any of the catalytic residues described as key for H_2_O_2_ enzymatic removal, a feature that was not observed among thousands of members identified for this superfamily. Arabidopsis APX-L, previously named as TL-29 (for thylakoid lumen 29 kDa protein) or APx04, was functionally and structurally investigated in the past [31,32,33]. Purification of native APX-L from chloroplasts extract showed that this protein accounts for one of the most abundant proteins in the thylakoid lumen of Arabidopsis, while recombinant expression showed that APX-L is not able to bind the heme, ascorbate, or catalyze H_2_O_2_ removal [32]. Interestingly, analyses conducted in Arabidopsis knockout plants suggest that APX-L is functional and participates in photosystem protection and seed coat formation. Mutants exhibit chlorotic phenotype and produce seeds with decreased longevity, but the mechanisms implicated in such phenotypes remain to be elucidated [33]. Despite the strong support for their reclassification as new families, APX-R and APX-L have been continuously addressed as ascorbate peroxidases in the literature, causing the misinterpretation of experimental data and impairing the discussion over H_2_O_2_ scavenging metabolism in plants. To understand how these three families are distributed among basal organisms in the plant lineage and to access the origin of the structural and functional diversity among them, we performed a comprehensive phylogenetic analysis using sequences retrieved from species of algae and bryophytes. Sequences that compose each cluster proved to be more heterogeneous than expected, and hybrid proteins, which combine key features from more than one family, are now described for the first time. The acknowledgment of this diversity provides the basis to explain the origin of APX-R and APX-L from ancestral ascorbate peroxidases and sheds light on unnoticed complexity in the hydrogen peroxidase metabolism of photosynthetic organisms.

## 2. Materials and Methods

### 2.1. Protein Sequence Retrieval

Publicly available protein sequences used in this study were retrieved from RedOxiBase [34]. For charophytes, the sequences were retrieved from the OneKP data (http://www.onekp.com/public_data.html, accessed on 1 May 2019) and the complete predicted proteome of *Klebsormidium nitens* (http://www.plantmorphogenesis.bio.titech.ac.jp/~algae_genome_project/klebsormidium/, accessed on 1 May 2019) using blastp searches (e-value < e^−5^) with the known *Arabidopsis thaliana* proteins were used as queries. Redundant sequences were eliminated using in-house scripts, keeping only the longest protein sequence. Sequences that were shorter than 50% of the Arabidopsis query were regarded as incomplete and discarded.

### 2.2. Sequence Alignment and Phylogenetic Analysis

Sequence alignments were conducted using MUSCLE algorithm [35] with default parameters available at MEGA 7.0 (Molecular Evolutionary Genetics Analysis) [36]. The phylogenetic tree was reconstructed using protein sequences of conserved domains by Bayesian inference using BEAST2.4.5 [37]. The best-fit model of amino acid replacement was LG with invariable sites and gamma-distributed rates, which was selected after analysis performed on ProtTest [38]. The Birth and Death Model was selected as tree prior, and 50,000,000 generations were performed with the Markov Chain Monte Carlo algorithm (MCMC) [39] for the evaluation of posterior distributions. After manual inspection of the alignment, 348 sequences and 233 sites were used in the analysis. Convergence was verified with Tracer [40], and the consensus tree was generated using TreeAnnotator, available at BEAST package. The resulting tree was analyzed and edited using FigTree v.1.4.3 (http://tree.bio.ed.ac.uk/software/figtree, accessed on 1 February 2020) and iTOL (https://itol.embl.de/, accessed on 1 August 2020). Figures that show sequence alignments were generated using Geneious Prime 2020.1.1 (https://www.geneious.com, accessed on 1 March 2020); for the gene structure figure, GSDS 2.0 was employed [41].

## 3. Results

### 3.1. Hybrid Proteins Share Features of Distinct Families

**Figure 1:**
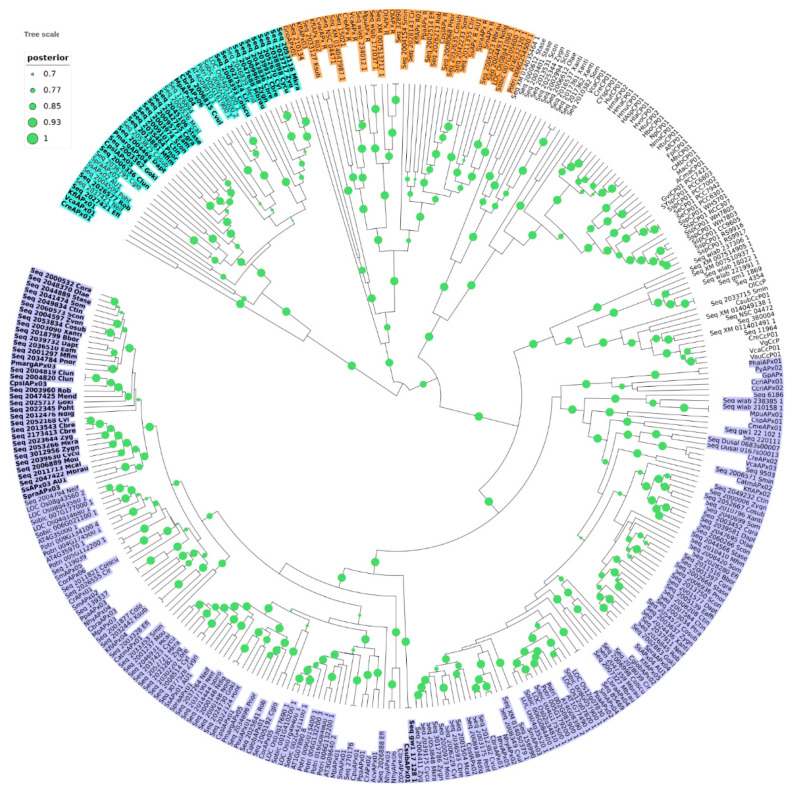
The phylogenetic relationship between APX, APX-R, APX-L, CCP, and KATG (CP) was reconstructed using the Bayesian method. A total of 348 protein sequences were included in the analysis, and ambiguous positions were removed from the alignment. APX, APX-R, and APX-L families are separated in three well-supported clusters and colored in purple (APX), orange (APX-R), and cyan (APX-L). Protein sequences that display hybrid features are indicated in bold letters. The posterior probabilities are discriminated according to the figure legend; only values above 0.7 are indicated.

Phylogenetic analysis was carried out with 309 protein sequences from species of algae and non-vascular plants, in addition to 39 sequences encoding non-plant KATG and CCP, which were used as outgroups. The dataset included 118 sequences previously deposited in RedOxiBase, annotated either as APX (which encompass both APX and misannotated APX-L) or APX-R, and 191 sequences from charophytes derived from 39 assembled transcriptomes and the complete genome of *Klebsormidium nitens*. From this analysis, it is possible to distinguish five well-supported main clusters, three of which correspond to APX (purple), APX-R (orange), and APX-L (cyan) families, in addition to KATG and CCP (Figure 1). The tree topology agrees with previous studies, showing that APX family is more closely related to KATG and CCP than to APX-R or APX-L (Figure 1), although being all part of the same peroxidase class [27]. A detailed analysis of each group revealed the existence of sequence variants, which are highlighted in Figure 1. These sequences consist of proteins that present characteristics that are singular of at least two different families (e.g., the simultaneous occurrence of Phe41, an APX-R/class II/class III feature, and Arg172, an APX specific residue), which was not observed previously. From now on, we will refer to these proteins as hybrids.

### 3.2. Hybrid Proteins Are More Prevalent in Species of Green Algae

**Figure 2:**
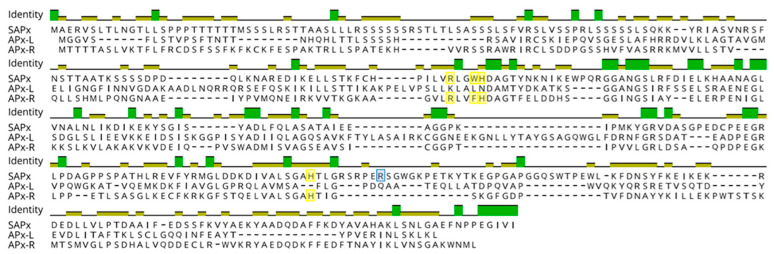
Protein sequence alignment of Arabidopsis stromal APX (SAPx; At4g08390), APX-R (At4g32320), and APX-L (At4g09010). Positions related to APX catalytic activity are highlighted in yellow and to ascorbate binding, in blue.

**Figure 3:**
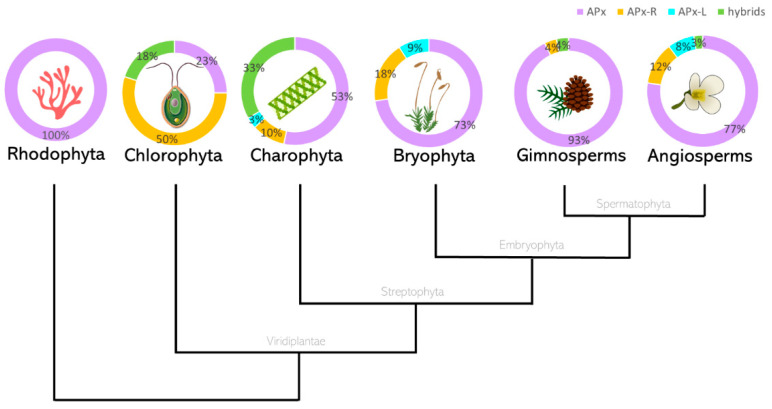
Protein sequences currently annotated in RedOxiBase were classified according to key residues as APX, APX-R, APX-L, or hybrids. The total number of sequences analyzed is 6, 22, 177, 11, 28, and 389, respectively. Each group of proteins is represented by the following colours: APX, purple; APX-R, orange; APX-L, cyan; and hybrids are shown in green. * The data presented for Charophytes include sequences currently annotated in RedOxiBase in addition to 147 assembled sequences obtained from transcriptomic data, available in Table S3.

To assess the prevalence of hybrid proteins in different groups of organisms, we decided to look for variants in APX, APX-L, and APX-R sequences deposited in public databases. For this purpose, 493 complete protein sequences were retrieved from RedOxiBase and further classified into rhodophytes, chlorophytes, charophytes, bryophytes, gymnosperms, and angiosperms. In addition, 143 charophytes sequences from our dataset were also evaluated, adding up to 636 sequences. The presence or absence of key residues implicated in catalytic activity and ascorbate binding determined their annotation, which followed the protein signature described for each family [27]. To be considered an APX, proteins should present the catalytic residues Arg38, Trp41, His42, and His163, in addition to the ascorbate-binding residue Arg172. For APX-R classification, proteins should include Arg38, Phe41, His42, and His163, and position 172 should be occupied by any residue other than arginine. To be classified as APX-L, all the above-mentioned positions should be occupied by other residues (Figure 2). Proteins were considered hybrids when they exhibited different arrangements of amino acids in these positions. The occurrence of each group of proteins in the analyzed classes is summarized in Figure 3, and their prevalence by species is provided in Table S1.

**Figure 4:**
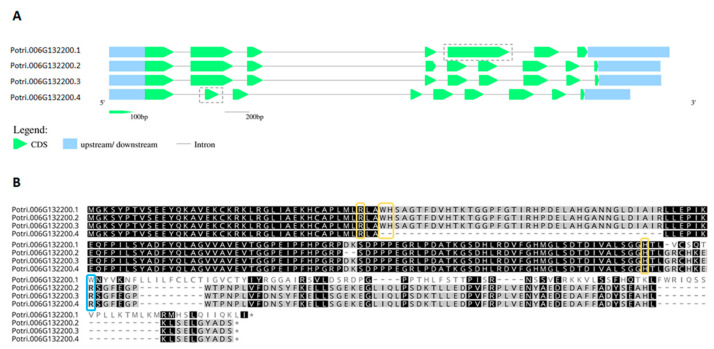
Alternative splicing favors the production of APX or hybrid proteins in *Populus trichocarpa*. (**A**). The gene Potri.006G132200 encodes four transcripts, two of which differ in the size of the second or fifth exons, indicated in the transcripts’ diagrams. (**B**). The retention of the truncated version of the second exon in Potri.006G132200.4 (PAC ID 27006574) causes the loss of two catalytic residues, which are essential for peroxidase activity (Trp41 and His42). In Potri.006G132200.1 (PAC ID 27006571), the alternative splicing produces a APX devoid of ascorbate binding site, in which Arg172 is replaced by a tryptophan. Ascorbate binding arginine is indicated in blue, and catalytic residues in yellow.

From this analysis, it is possible to infer that the diversification of APX must have occurred at the basis of Viridiplantae. However, one must consider that the low number of sequences currently available from rhodophytes could be leading to a biased interpretation in this subject. In Viridiplantae, hybrid proteins are considerably more prevalent in organisms belonging to deep-branching clades, accounting for up to 33% of total analyzed sequences derived from charophytes. While APX-R is present in all the analyzed classes of Viridiplantae, APX-L could only be detected in charophytes, bryophytes, and angiosperms, in rates of 3% to 9%. Among the examined groups, gymnosperms are the organisms with the higher prevalence of APX proteins (93%), followed by angiosperms. All complete protein sequences deposited in RedOxiBase and classified as APX-R, APX-L, or hybrid are listed in Table S2. Most of these proteins are predicted to be imported to chloroplasts, but there are also a few that seem to target other subcellular compartments (Table S2) [42,43,44]. Although hybrid proteins are more prevalent in basal organisms, which is partly explained by the late expansion of the APX family in land plants (Table S1), some can be found in vascular plants. An interesting example is observed in poplar (*Populus trichocarpa*), in which a single gene encodes an APX and two hybrid proteins through alternative splicing, producing a variant that potentially retained the ability to bind ascorbate while deprived of peroxidatic activity, and a peroxidase devoid of ascorbate binding site (Figure 4). The production of distinguished APX isoforms through alternative splicing was observed previously for spinach (*Spinacia oleracea*), which suggests that this could be a conserved mechanism of APX regulation [45].

### 3.3. Mutations and Small Deletions Led to the Emergence of APX-R and APX-L

**Figure 5:**
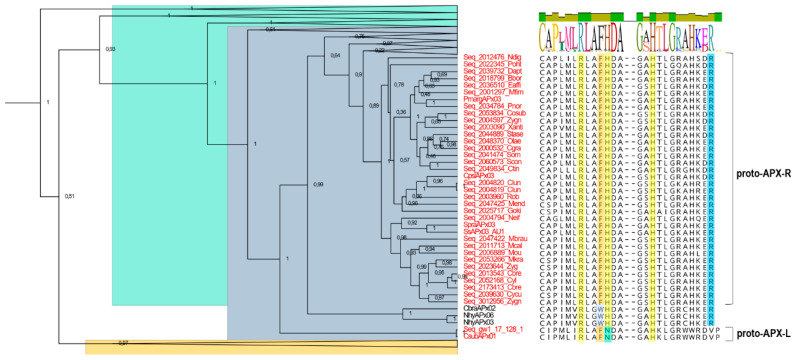
Detailed analysis of a clade from the APX cluster. Hybrid sequences derived from Charophytes that exhibit a single mutation from tryptophan to phenylalanine at position 41 are addressed as proto-APX-R. The loss of arginine at position 172 and the replacement of the distal histidine by an asparagine illustrate the diversification pathway towards APX-L establishment, for which reason these sequences are referred to as proto-APX-L. APX group is shown in blue; APX-R, in orange; and APX-L, in green. All other clades showed in Figure 1 were collapsed for better visualization. Sequence logo presented above the alignment shows a consensus sequence of all listed species.

A large proportion of the hybrid proteins identified in this study is organized in a cluster that integrates the well-supported APX group in the phylogenetic analysis, presented on Figure 1. Despite the overall similarity with APX, these charophyte sequences contain a phenylalanine at position 41 instead of the APX-conserved tryptophan, which suggests that this mutation could have been implicated with the first modifications that accompanied the establishment of APX-R (Figure 5). Similarly, two charophyte sequences that also integrate the APX group exhibit an arrangement of residues that might help explaining the evolution of APX-L. These sequences also contain phenylalanine at position 41, while having lost the distal histidine and the ascorbate-binding arginine. These two types of sequences are here named as proto-APX-R and proto-APX-L, respectively, and their identification provides evidence for the establishment of APX-R and APX-L families from ancestral APX diversification.

**Figure 6:**
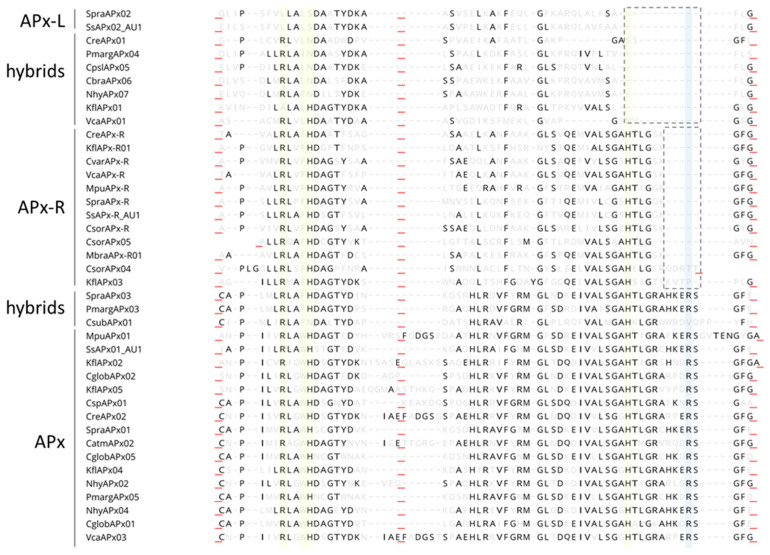
Mutations and two small deletions are involved with APX-R and APX-L establishment. Representative sequences of each protein category were retrieved from RedOxiBase, and domains that contain relevant residues are shown. Conserved residues are shown in black and others, in light grey. Positions implicated with catalytic activity are shown in yellow and with ascorbate-binding, in blue. While mutations seem to be responsible for the progressive loss of N-terminal catalytic residues, two small deletions appear to be the main cause leading to Arg172 and His163 loss. The alignment is shown from protein position 32. Red underscores evidence of domains collapsing for better visualization.

To analyze the extent of variation found in these distinct groups of proteins, we performed an alignment using representative sequences from chlorophytes and charophytes, which is presented in Figure 6. Through this analysis, it is possible to observe that several mutations and two small deletions around the proximal histidine are the main cause of variation found in these families regarding catalytic and ascorbate-binding residues. This analysis also suggests that hybrid proteins accumulated fewer mutations than APX-R and APX-L, resembling intermediates among such peroxidase families.

## 4. Discussion

The structure and function of APX were extensively studied since recombinant protein expression and site-directed mutagenesis techniques were established. Due to its overall homology to CCP, APX was expected to display a similar enzymatic activity. However, because APX catalysis relies on the formation of a porphyrin-based radical, and not on a protein-based radical (which is the case for CCP), APX quickly became an interesting model to dissect heme peroxidase catalysis in non-animal organisms [46]. Since the publication of APX first crystal structure APX, and later of APX-ascorbate complex structure, all main residues implicated in the enzymatic activity displayed towards this substrate were identified [15]. Site-directed mutagenesis have later confirmed that two histidines (His42 and His163), an arginine (Arg38), and a tryptophan (Trp41) are essential for APX to catalyze H_2_O_2_ scavenging. Apart from Trp41, which was successfully substituted by phenylalanine in proteins belonging to the late-emerging classes II and III [47], all other residues are conserved and proved to be critical for superfamily peroxidases to interact with the heme moiety and with H_2_O_2_. Regarding APX interaction with its substrate, despite the evidence that Lys30 and Cys32 could be involved, an arginine at position 172 has proved to be the critical residue for APX to bind ascorbate [9,10]. Meanwhile, Cys32 was shown to undergo S-nitrosation, a post-translational modification with a positive effect on APX activity [48]. While substitutions at Arg172 abolished APX activity towards this substrate, they did not interfere with APX binding to non-physiological aromatic substrates in vitro, indicating that APX could also interact with other molecules in vivo through distinct sites [13,14,49]. Recently, a study demonstrated that cytosolic APX catalyzes the hydroxylation of 4-coumarate to caffeate in lignin biosynthesis, in a reaction dependent on ascorbate and molecular oxygen [50]. Through this study, the physiological relevance of this distinguished substrate binding site through which APX can interact with other phenolic compounds was finally confirmed.

Because Arg172 is missing in APX-R, we previously suggested that members of this family should display peroxidase activity using other substrates than ascorbate, which was recently confirmed through heterologous expression and enzymatic assays performed in vitro [30]. Despite differences in substrate, plastidial APX and APX-R exhibit comparable peroxidase activity. Both peroxidases are able to interact with pyrogallol and guaiacol in vitro at similar rates, showing the preservation of one substrate binding site. Interestingly, this is not the case for APX-L. It was previously reported that Arabidopsis APX-L is devoid of catalytic activity (as a peroxidase) and unable to bind ascorbate or the heme, although being expressed at high levels and somehow functional, with knockout plants showing a chlorotic phenotype and producing seeds with reduced longevity [31,32,33]. In addition, the authors showed experimental evidence of APX-L association with the photosystem II, supporting a divergent role for this protein. A large recent study, by using co-fractionation mass spectrometry, determined protein complexes from several plant species, including Arabidopsis [51]. Through the data generated in this study, several candidate proteins that might be interacting with APX-L in this species were identified, among them proteins directly involved in the photosystem II redox status. Among the candidates with the highest confidence scores, photosystem II subunits Q and P, and peroxiredoxin Q, are listed (Table S4). A curious observation is that most APX-L found in the Angiosperms exhibit conserved leucine and asparagine at positions 41 and 42, while in deep-branching clades no particular conservation could be observed in these and other positions considered key for the superfamily.

**Figure 7:**
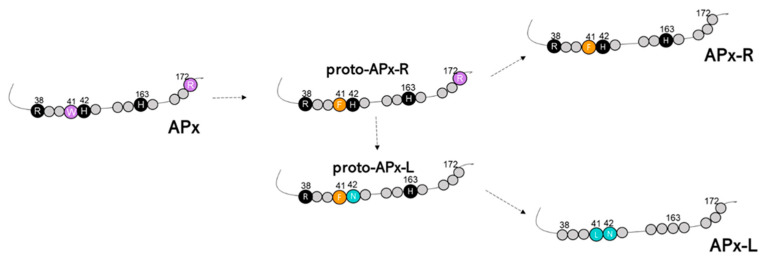
The proposed model to explain the origin of APX-R and APX-L. Relevant residues are represented based on angiosperms sequences, and their position is discriminated. Conserved amino acids are indicated and coloured according to the group they are primarily found. Amino acid numbering refers to cytosolic ascorbate peroxidase from *Pisum sativum* (NCBI accession P48534).

It is believed that the peroxidase-catalase superfamily, which is composed of 12 families of peroxidases accommodated in three classes, is derived from an ancestral peroxidase that resembled the bifunctional catalase-peroxidase known as KATG and had all conserved positions in the active center mentioned above [25]. The superfamily emergence is explained as the result of countless duplication events, and it seems to be tightly associated with mitochondria and chloroplast acquisitions. Because of this, all superfamily enzymes share common characteristics, like rather conserved structure and folding, despite divergencies observed in amino acid composition [15]. It is also likely that the accumulation of spontaneous natural mutations eventually culminated in the establishment of the families that we encounter nowadays. Additionally, the occurrence of APX-CCP proteins in some species and the functional diversity observed in such proteins provide strong support for this model [52,53]. In this scenario, the identification of hybrid proteins is another outstanding piece of evidence of this evolutionary process. For being more closely related to bifunctional bacterial KATG and other class I enzymes, APX is shown here to be more ancestral than APX-R and APX-L. We hypothesize that the acquisition of new functions must have been accompanied by the progressive loss of the ancestral and eventually obsolete ascorbate-binding site in APX-R, and of the catalytic sites in APX-L, which are evidenced by the identification of proto APX-R, proto APX-L, and hybrids. Besides, other residues implicated in APX activity regulation (e.g., Cys32) also diverged in members of APX-R and APX-L families (Figure 6). The analyses carried out in this study indicate that cumulative mutations and small deletions might have been the main driving force behind APX-R and APX-L establishment in chlorophyte and charophyte ancestors, respectively. We therefore propose a model for the emergence of these proteins in autotrophic eukaryotes, which is presented in Figure 7.

The absence of typical APX-L in chlorophytes indicates that this group of proteins was established more recently in a Streptophyta ancestor; however, this hypothesis remains to be confirmed when genomic and transcriptomic data from other species that compose this group become available. Nevertheless, the amino acid arrangement observed in proto-APX-R and proto-APX-L, and the number of mutations that were necessary for the establishment of both families, support an earlier divergence of APX-R. The dissimilar distribution of each family and hybrid proteins in the analyzed groups also suggests that this process occurred under distinct selective pressures in aquatic and terrestrial organisms. Nevertheless, the maintenance of hybrid proteins must have been advantageous to some plants, as in the case of poplar. Despite the limited number of sequences from basal organisms, an interesting observation is that a few algae species contain more than one APX-R encoding gene, in contrast to what we observed previously for vascular plants, in which in genomes the duplication of this gene seems to be detrimental [28].

The functionality of APX-R, APX-L, and hybrid proteins is yet unclear. While it is now confirmed that APX-R displays significantly modified peroxidase activity, the lack of information regarding its natural substrate precludes our understanding of the metabolic pathways in which this enzyme participates. However, the studies made so far indicate that this peroxidase acts on seed redox and hormonal metabolism, developmental and stress-induced senescence, and photomorphogenesis [29,30,54], which could indicate starting points for substrate identification. In the case of APX-L, evidence suggest that members of this family could act as modulating proteins redox status via protein-protein interaction; therefore, the search for interaction partners in combination with global analyses of transcripts and/or proteins could be a solid strategy for its evaluation. The characterization of APX-R and APX-L function in vivo will provide the basis for us to understand which residues are key for their specific functions, and how hybrids behave in this context. Even though hybrids do not characterize as distinct families, these proteins indicate that there might be more plasticity in the peroxidase metabolism than previously believed.

## 5. Conclusions

This work provides new evidence on the origin and evolution of APX, APX-R, and APX-L families in the plant lineage and reveals the existence of significant diversity in the complex peroxidase-catalase superfamily, which should be taken into consideration when assessing heme peroxidases function in photosynthetic organisms.

## Data Availability

Data is contained within the article or Supplementary Material.

## Supplementary Materials

The following are available online at https://www.mdpi.com/article/10.3390/antiox10040597/s1, Table S1: Occurrence of APX, APX-R, APX-L and hybrid proteins in species of Archeaplastida based on sequences currently deposited in RedOxiBase and OneKP, Table S2: Accession numbers of APX-R, APX-L and hybrid proteins deposited in RedOxiBase, Table S3: Charophytes sequences retrieved from OneKP used in the analyses, Table S4: APX-L protein-protein interactions deposited on plant.MAP database for Arabidopsis thaliana (http://plants.proteincomplexes.org/).
