# Supplementary material for: Ascorbate Peroxidase Neofunctionalization at the Origin of APX-R and APX-L: Evidence from Basal Archaeplastida
Source: Antioxidants (Basel). 2021 Apr 13;10(4):597. doi: 10.3390/antiox10040597 (PMC8069737; doi:10.3390/antiox10040597)
Supplement: Supplementary file 1 [file antioxidants-10-00597-s001.zip › Supplementary material tables S1 S2 S4 revised 2.docx]

**Table S1**. Occurrence of APx, APx-R, APx-L and hybrid proteins in species of Archeaplastida based on sequences currently deposited in RedOxiBase and OneKP

|  | **APx** | **APx-R** | **APx-L** | **hybrid** |
| --- | --- | --- | --- | --- |
| **Rhodophyta** |  |  |  |  |
| *Cyanidioschyzon merolae* | 1 | 0 | 0 | 0 |
| *Galdieria partita* | 1 | 0 | 0 | 0 |
| *Galdieria sulphuraria* | 2 | 0 | 0 | 0 |
| *Porphyra raitanensis* | 1 | 0 | 0 | 0 |
| *Porphyra yezoensis* | 1 | 0 | 0 | 0 |
|  |  |  |  |  |
| **Chlorophyta** |  |  |  |  |
| *Chlamydomonas reinhardtii* | 1 | 1 | 0 | 1 |
| *Chlorella sorokiniana* | 1 | 5 | 0 | 0 |
| *Chlamydomonas sp* | 1 | 0 | 0 | 0 |
| *Coccomyxa subellipsoidea* | 0 | 0 | 0 | 1 |
| *Chlorella variabilis (C.sp. NC64A)* | 0 | 1 | 0 | 0 |
| *Micromonas pusilla* | 1 | 1 | 0 | 0 |
| *Ostreococcus lucimarinus* | 0 | 1 | 0 | 0 |
| *Ostreococcus tauri* | 1 | 1 | 0 | 0 |
| *Volvox carteri* | 1 | 1 | 0 | 1 |
|  |  |  |  |  |
| **Charophyta** |  |  |  |  |
| *Chlorokybus atmophyticus* | 3 | 1 | 0 | 0 |
| *Chara braunii* | 2 | 0 | 0 | 1 |
| *Chaetosphaeridium globosum* | 3 | 0 | 0 | 0 |
| *Closterium peracerosum-strigosum-littorale* | 0 | 0 | 0 | 1 |
| *Klebsormidium flaccidum* | 3 | 3 | 0 | 1 |
| *Mesotaenium braunii* | 0 | 1 | 0 | 0 |
| *Nitella hyalina* | 5 | 0 | 0 | 1 |
| *Penium margaritaceum* | 1 | 0 | 0 | 2 |
| *Spirogyra pratensis* | 1 | 1 | 1 | 1 |
| *Spirogyra sp* | 2 | 1 | 1 | 1 |
| *Entransia fimbriat* | 3 | 1 | 0 | 1 |
| *Cosmarium tinctum* | 3 | 1 | 0 | 2 |
| *Desmidium aptogonum* | 2 | 0 | 0 | 2 |
| *Closterium lunula* | 0 | 0 | 0 | 3 |
| *Chaetosphaeridium globosum* | 2 | 0 | 1 | 0 |
| *Netrium digitus* | 3 | 1 | 0 | 1 |
| *Klebsormidium subtile* | 1 | 1 | 0 | 0 |
| *Xanthidium antilopaeum* | 2 | 0 | 0 | 1 |
| *Onychonema laeve* | 1 | 0 | 0 | 1 |
| *Euastrum affine* | 1 | 0 | 0 | 2 |
| *Mesotaenium caldariorum* | 2 | 0 | 1 | 1 |
| *Staurastrum sebaldi* | 2 | 0 | 0 | 2 |
| *Cylindrocystis cushleckae* | 2 | 0 | 0 | 2 |
| *Gonatozygon kinahanii* | 2 | 0 | 0 | 2 |
| *Nucleotaenium eifelense* | 3 | 0 | 1 | 1 |
| *Micrasterias fimbriata* | 1 | 0 | 0 | 2 |
| *Zygnemopsis sp.* | 2 | 0 | 0 | 2 |
| *Cosmarium granatum* | 2 | 0 | 0 | 2 |
| *Pleurotaenium trabecul* | 1 | 0 | 0 | 0 |
| *Chara vulgaris* | 2 | 0 | 0 | 1 |
| *Mesotaenium kramstei* | 2 | 0 | 0 | 2 |
| *Spirotaenia minuta* | 2 | 1 | 0 | 0 |
| *Coleochaete irregularis* | 2 | 1 | 0 | 1 |
| *Bambusina borreri* | 1 | 0 | 0 | 1 |
| *Phymatodocis nordstedtiana* | 2 | 1 | 0 | 2 |
| *Staurodesmus omearii* | 2 | 1 | 0 | 1 |
| *Planotaenium ohtanii* | 2 | 0 | 1 | 1 |
| *Zygnema sp.-A* | 2 | 0 | 0 | 1 |
| *Spirotaenia sp.* | 1 | 0 | 0 | 0 |
| *Cylindrocystis sp.* | 2 | 0 | 0 | 2 |
| *Coleochaete scutata* | 2 | 0 | 0 | 1 |
| *Staurodesmus convergens* | 2 | 0 | 0 | 2 |
| *Mesotaenium endlicherianum* | 0 | 0 | 0 | 2 |
| *Cosmarium subtumidum* | 3 | 1 | 0 | 1 |
| *Zygnema sp.-B* | 2 | 0 | 0 | 2 |
| *Mesotaenium braunii* | 2 | 1 | 0 | 2 |
| *Roya obtusa* | 2 | 1 | 0 | 2 |
| *Cylindrocystis brebissonii* | 2 | 0 | 0 | 4 |
| *Mougeotia sp.* | 2 | 0 | 0 | 1 |
|  |  |  |  |  |
| **Bryophyta** |  |  |  |  |
| *Marchantia paleacea* | 1 | 0 | 0 | 0 |
| *Nothoceros aenigmaticus* | 1 | 0 | 0 | 0 |
| *Physcomitrella patens* | 3 | 1 | 0 | 0 |
| *Marchantia polymorpha* | 3 | 1 | 0 | 1 |
|  |  |  |  |  |
| **Gymnosperms** |  |  |  |  |
| *Pinus pinaster* | 2 | 0 | 0 | 0 |
| *Cycas rumphii* | 2 | 0 | 0 | 0 |
| *Welwitschia mirabilis* | 2 | 0 | 0 | 0 |
| *Zamia fischeri* | 2 | 0 | 0 | 0 |
| *Picea glauca* | 2 | 0 | 0 | 0 |
| *Picea sitchensis* | 2 | 0 | 0 | 0 |
| *Cryptomeria japonica* | 2 | 0 | 0 | 0 |
| *Pinus taeda* | 5 | 0 | 0 | 0 |
| *Ginkgo biloba* | 4 | 1 | 0 | 1 |
| *Picea abies* | 3 | 0 | 0 | 0 |
| *Wollemia nobilis* | 1 | 0 | 0 | 0 |
|  |  |  |  |  |
| **Monocots** |  |  |  |  |
| *Acorus americanus* | 1 | 0 | 0 | 0 |
| *Allium cepa* | 3 | 0 | 0 | 0 |
| *Asparagus officinalis* | 1 | 0 | 0 | 0 |
| *Brachypodium distachyon* | 7 | 1 | 1 | 0 |
| *Crocus sativus* | 1 | 0 | 0 | 0 |
| *Elaeis guineensis* | 2 | 0 | 0 | 0 |
| *Festuca arundinacea* | 0 | 1 | 0 | 0 |
| *Hordeum vulgare* | 7 | 1 | 0 | 0 |
| *Oryza brachyantha* | 0 | 1 | 0 | 0 |
| *Oryza sativa ssp japonica cv Nipponbare* | 9 | 1 | 1 | 0 |
| *Oryza sativa (indica cultivar-group)* | 0 | 1 | 0 | 0 |
| *Pennisetum americanum* | 1 | 0 | 0 | 0 |
| *Sorghum bicolor* | 7 | 1 | 1 | 0 |
| *Secale cereal* | 1 | 0 | 0 | 0 |
| *Setaria italica* | 7 | 1 | 1 | 0 |
| *Spirodela polyrhiza* | 5 | 0 | 1 | 1 |
| *Triticum aestivum* | 7 | 1 | 0 | 0 |
| *Triticum monococcum* | 1 | 0 | 0 | 0 |
| *Zantedeschia aethiopica* | 3 | 0 | 0 | 0 |
| *Zostera marina* | 4 | 1 | 1 | 1 |
| *Zea mays* | 9 | 1 | 1 | 0 |
|  |  |  |  |  |
| **Eudicots** |  |  |  |  |
| *Aquilegia coerulea* | 2 | 1 | 0 | 0 |
| *Aquilegia formosa x Aquilegia pubescens* | 4 | 1 | 0 | 0 |
| *Arabidopsis halleri* | 0 | 1 | 0 | 0 |
| *Arabidopsis lyrata* | 8 | 1 | 1 | 0 |
| *Arabidopsis thaliana* | 6 | 1 | 1 | 0 |
| *Arachis hypogaea* | 2 | 0 | 0 | 0 |
| *Boechera stricta* | 2 | 0 | 0 | 0 |
| *Brassica juncea* | 2 | 0 | 0 | 0 |
| *Brassica napus* | 4 | 1 | 0 | 0 |
| *Brassica oleracea* | 2 | 1 | 0 | 0 |
| *Brassica rapa* | 2 | 1 | 0 | 0 |
| *Camellia sinensis* | 1 | 0 | 0 | 0 |
| *Capsella grandiflora* | 6 | 1 | 1 | 0 |
| *Capsicum annuum* | 4 | 0 | 0 | 0 |
| *Carica papaya* | 3 | 0 | 0 | 0 |
| *Catharanthus roseus* | 4 | 1 | 0 | 0 |
| *Cichorium intybus* | 1 | 0 | 0 | 0 |
| *Citrus clementina* | 5 | 1 | 1 | 0 |
| *Citrus sinensis* | 5 | 1 | 0 | 0 |
| *Codonopsis lanceolata* | 1 | 0 | 0 | 0 |
| *Cucumis melo* | 1 | 0 | 0 | 0 |
| *Cucumis sativus* | 5 | 1 | 1 | 0 |
| *Cucurbita cv. Kurokawa Amakuri* | 3 | 0 | 0 | 0 |
| *Eucalyptus camaldulensis* | 3 | 1 | 0 | 0 |
| *Eucalyptus globulus* | 6 | 1 | 1 | 0 |
| *Eucalyptus grandis* | 6 | 1 | 1 | 0 |
| *Eucalyptus gunnii* | 6 | 1 | 1 | 0 |
| *Fragaria x ananassa* | 3 | 0 | 0 | 0 |
| *Gerbera hybrida cv. Terra Regina* | 2 | 0 | 0 | 0 |
| *Glycine max* | 7 | 1 | 2 | 1 |
| *Gossypium hirsutum* | 2 | 1 | 0 | 0 |
| *Gossypium raimondii* | 3 | 1 | 0 | 0 |
| *Hedyotis centranthoides* | 1 | 0 | 0 | 0 |
| *Hedyotis terminalis* | 1 | 0 | 0 | 0 |
| *Helianthus annuus* | 5 | 1 | 0 | 0 |
| *Helianthus argophyllus* | 2 | 0 | 0 | 0 |
| *Helianthus paradoxus* | 1 | 0 | 0 | 0 |
| *Hevea brasiliensis* | 2 | 0 | 0 | 0 |
| *Ipomoea batatas* | 1 | 0 | 0 | 0 |
| *Ipomoea nil* | 2 | 0 | 0 | 0 |
| *Lactuca sativa* | 2 | 0 | 0 | 1 |
| *Linum usitatissimum* | 5 | 1 | 2 | 1 |
| *Lotus japonicus (corniculatus var japonicus)* | 2 | 0 | 0 | 0 |
| *Lycopersicon esculentum* | 8 | 1 | 1 | 1 |
| *Malus domestica* | 2 | 1 | 0 | 0 |
| *Manihot esculenta* | 7 | 1 | 1 | 0 |
| *Medicago truncatula* | 5 | 1 | 1 | 0 |
| *Mesembryanthemum crystallinum* | 4 | 2 | 0 | 1 |
| *Mimulus guttatus* | 4 | 1 | 1 | 0 |
| *Nicotiana tabacum* | 6 | 0 | 2 | 1 |
| *Pimpinella brachycarpa* | 1 | 0 | 0 | 0 |
| *Pisum sativum* | 1 | 0 | 0 | 0 |
| *Poncirus trifoliata* | 2 | 0 | 0 | 0 |
| *Populus balsamifera* | 3 | 0 | 0 | 0 |
| *Populus tomentosa* | 1 | 0 | 0 | 0 |
| *Populus trichocarpa* | 8 | 1 | 1 | 0 |
| *Prunus persica* | 5 | 1 | 1 | 0 |
| *Quercus shumardii* | 1 | 0 | 0 | 0 |
| *Raphanus sativus* | 1 | 0 | 0 | 0 |
| *Rehmannia glutinosa* | 1 | 0 | 0 | 0 |
| *Retama raetam* | 1 | 0 | 0 | 0 |
| *Rheum australe* | 1 | 0 | 0 | 0 |
| *Ricinus communis* | 2 | 1 | 0 | 2 |
| *Solanum tuberosum* | 5 | 0 | 0 | 0 |
| *Spinacia oleracea* | 3 | 0 | 0 | 1 |
| *Suaeda salsa* | 1 | 0 | 0 | 0 |
| *Tarenaya hassleriana* | 0 | 1 | 0 | 0 |
| *Thellungiella parvula* | 1 | 0 | 1 | 0 |
| *Thellungiella salsuginea* | 9 | 1 | 0 | 0 |
| *Vigna unguiculata* | 4 | 0 | 0 | 0 |
| *Vitis hybrid cultivar* | 1 | 0 | 0 | 0 |
| *Vitis vinifera* | 4 | 2 | 1 | 0 |

| Table S2. Accession numbers of APx-R, APx-L and hybrid proteins deposited in RedOxiBase | | | | | | |  |
| --- | --- | --- | --- | --- | --- | --- | --- |
|  | **Classification** | **RedOxiBase ID** | **Organism** | **ChloroP ^[52]^** | **LOCALIZER ^[53]^** | **WOLF PSORT ^[54]^** |  |
| Chlorophyta | APx-R | 7433 | *Chlamydomonas reinhardtii* | chloroplast | chloroplast | chloroplast |  |
|  |  | 7453 | *Ostreococcus lucimarinus* | chloroplast | chloro/mito | chloroplast |  |
|  |  | 8358 | *Ostreococcus tauri* | chloroplast | chloroplast | mitochondria |  |
|  |  | 15183 | *Chlorella sorokiniana* | chloroplast | chloroplast | chloroplast |  |
|  |  | 15185 | *Chlorella sorokiniana* | - | nucleus | plasma memb |  |
|  |  | 15186 | *Chlorella sorokiniana* | - | nucleus | extracellular |  |
|  |  | 15187 | *Chlorella sorokiniana* | - | - | extracellular |  |
|  |  | 15188 | *Chlorella sorokiniana* | - | - | extracellular |  |
|  |  | 8356 | *Chlorella variabilis* | chloroplast | chloro/nucleus | chloroplast |  |
|  |  | 8357 | *Volvox carteri* | - | - | plasma memb |  |
|  |  | 8359 | *Micromonas pusilla* | chloroplast | chloroplast | chloroplast |  |
|  | hybrid | 13010 | *Bathycoccus prasinos* | - | nucleus | mitochondria |  |
|  |  | 2805 | *Chlamydomonas reinhardtii* | chloroplast | chloro/mito | chloroplast |  |
|  |  | 11186 | *Coccomyxa subellipsoidea* | - | nucleus | cytosol |  |
|  |  | 8560 | *Volvox carteri* | chloroplast | mito/nucleus | chloroplast |  |
| Charophyta | APx-R | 12574 | *Spirogyra sp* | - | nucleus | chloroplast |  |
|  |  | 11245 | *Spirogyra pratensis* | - | - | chloroplast |  |
|  |  | 7736 | *Klebsormidium flaccidum* | chloroplast | - | cytosol |  |
|  |  | 10052 | *Klebsormidium flaccidum* | - | - | chloroplast |  |
|  |  | 376 | *Klebsormidium flaccidum* | chloroplast | - | chloroplast |  |
|  |  | 7707 | *Chlorokybus atmophyticus* | chloroplast | chloroplast | extracellular |  |
|  |  | 15548 | *Mesotaenium braunii* | - | - | chloroplast |  |
|  | APx-L | 12582 | *Spirogyra sp* | chloroplast | chloro/nucleus | chloroplast |  |
|  |  | 7756 | *Spirogyra pratensis* | chloroplast | chloroplast | chloroplast |  |
|  | hybrid | 7766 | *Penium margaritaceum* | chloroplast | chloroplast | chloroplast |  |
|  |  | 12940 | *Closterium peracerosum-strigosum-littorale* | chloroplast | chloroplast | chloroplast |  |
|  |  | 15179 | *Nitella hyalina* | chloroplast | chloroplast | chloroplast |  |
|  |  | 12967 | *Chara braunii* | chloroplast | chloro/mito/nuc | chloroplast |  |
|  |  | 12584 | *Spirogyra sp* | - | nucleus | cytosol |  |
|  |  | 7758 | *Spirogyra pratensis* | - | nucleus | cytosol |  |
|  |  | 7754 | *Penium margaritaceum* | - | - | cytosol |  |
|  |  | 7731 | *Klebsormidium flaccidum* | chloroplast | chloroplast | chloroplast |  |
| Bryophytes | APx-R | 5761 | *Physcomitrella_patens* | chloroplast | chloroplast | chloroplast |  |
|  |  | 5764 | *Marchantia polymorpha* | chloroplast | chloroplast | chloroplast |  |
|  | hybrid | 7344 | *Marchantia polymorpha* | chloroplast | chloroplast | chloroplast |  |
| Gymnosperms | APx-R | 13128 | *Ginkgo biloba* | - | nucleus | chloroplast |  |
|  | hybrid | 13085 | *Ginkgo biloba* | chloroplast | chloroplast | chloroplast |  |
| Monocots | APx-R | 7459 | *Brachypodium distachyon* | chloroplast | chloroplast | chloroplast |  |
|  |  | 5177 | *Festuca arundinacea* | chloroplast | chloroplast | chloroplast |  |
|  |  | 5184 | *Hordeum vulgare* | chloroplast | chloroplast | chloroplast |  |
|  |  | 11361 | *Oryza brachyantha* | chloroplast | chloroplast | chloroplast |  |
|  |  | 3961 | *Oryza sativa ssp japonica* | chloroplast | chloroplast | chloroplast |  |
|  |  | 7156 | *Oryza sativa ssp indica* | chloroplast | chloroplast | chloroplast |  |
|  |  | 5185 | *Sorghum bicolor* | - | nucleus | plasma memb |  |
|  |  | 8369 | *Setaria italica* | chloroplast | chloroplast | chloroplast |  |
|  |  | 13237 | *Spirodela polyrhiza* | chloroplast | chloro/nucleus | chloroplast |  |
|  |  | 5193 | *Triticum aestivum* | chloroplast | chloroplast | chloroplast |  |
|  |  | 10861 | *Zostera marina* | - | - | chloroplast |  |
|  |  | 5183 | *Zea mays* | chloroplast | chloroplast | chloroplast |  |
|  | APx-L | 7925 | *Brachypodium distachyon* | chloroplast | - | chloroplast |  |
|  |  | 3966 | *Oryza sativa ssp japonica* | chloroplast | chloroplast | chloroplast |  |
|  |  | 7999 | *Sorghum bicolor* | chloroplast | chloroplast | chloroplast |  |
|  |  | 9433 | *Setaria italica* | chloroplast | chloro/mito | chloroplast |  |
|  |  | 13227 | *Spirodela polyrhiza* | - | - | extracellular |  |
|  |  | 6708 | *Zea mays* | chloroplast | chloroplast | chloroplast |  |
|  |  | 14371 | *Zostera marina* | chloroplast | chloroplast | chloroplast |  |
|  | hybrid | 13246 | *Spirodela polyrhiza* | - | - | - |  |
|  |  | 14364 | *Zostera marina* | - | - | cytosol |  |
| Eudicots | APx-R | 2495 | *Mesembryanthemum crystallinum* | - | - | cytosol |  |
|  |  | 2492 | *Mesembryanthemum crystallinum* | - | - | cytosol |  |
|  |  | 3952 | *Arabidopsis thaliana* | chloroplast | chloroplast | chloroplast |  |
|  |  | 5173 | *Vitis vinifera* | chloroplast | chloroplast | chloroplast |  |
|  |  | 5179 | *Aquilegia formosa x Aquilegia pubescens* | chloroplast | chloroplast | chloroplast |  |
|  |  | 5180 | *Gossypium raimondii* | chloroplast | chloroplast | chloroplast |  |
|  |  | 5181 | *Brassica napus* | chloroplast | chloroplast | chloroplast |  |
|  |  | 5182 | *Glycine max* | chloroplast | - | chloroplast |  |
|  |  | 5186 | *Lycopersicon esculentum* | chloroplast | chloroplast | chloroplast |  |
|  |  | 5191 | *Malus domestica* | chloroplast | chloroplast | chloroplast |  |
|  |  | 5192 | *Populus trichocarpa* | chloroplast | chloroplast | chloroplast |  |
|  |  | 5198 | *Medicago truncatula* | chloroplast | chloro/mito | mitochondria |  |
|  |  | 5759 | *Gossypium hirsutum* | chloroplast | chloroplast | chloroplast |  |
|  |  | 7460 | *Manihot esculenta* | chloroplast | chloro/nucleus | chloroplast |  |
|  |  | 7461 | *Cucumis sativus* | chloroplast | chloroplast | chloroplast |  |
|  |  | 7462 | *Arabidopsis lyrata* | chloroplast | chloroplast | peroxisome |  |
|  |  | 8079 | *Eucalyptus grandis* | chloroplast | chloroplast | chloroplast |  |
|  |  | 8353 | *Brassica oleracea* | chloroplast | chloroplast | chloroplast |  |
|  |  | 8354 | *Brassica rapa* | chloroplast | chloroplast | chloroplast |  |
|  |  | 8364 | *Mimulus guttatus* | chloroplast | chloroplast | chloroplast |  |
|  |  | 8365 | *Ricinus communis* | - | chloroplast | chloroplast |  |
|  |  | 8367 | *Citrus sinensis* | chloroplast | chloro/nucleus | chloroplast |  |
|  |  | 8436 | *Eucalyptus globulus* | chloroplast | chloroplast | chloroplast |  |
|  |  | 8448 | *Aquilegia coerulea* | chloroplast | chloroplast | chloroplast |  |
|  |  | 8795 | *Citrus clementina* | chloroplast | chloro/nucleus | chloroplast |  |
|  |  | 9410 | *Vitis vinifera* | - | nucleus | nucleus |  |
|  |  | 9804 | *Thellungiella salsuginea* | chloroplast | chloroplast | chloroplast |  |
|  |  | 9945 | *Prunus persica* | chloroplast | chloroplast | chloroplast |  |
|  |  | 10235 | *Eucalyptus camaldulensis* | chloroplast | chloroplast | chloroplast |  |
|  |  | 11167 | *Helianthus annuus* | chloroplast | chloroplast | chloroplast |  |
|  |  | 11378 | *Eucalyptus gunnii* | chloroplast | chloroplast | chloroplast |  |
|  |  | 12008 | *Linum usitatissimum* | chloroplast | chloro/mito | chloroplast |  |
|  |  | 13328 | *Catharanthus roseus* | - | - | nucleus |  |
|  |  | 14777 | *Arabidopsis halleri* | chloroplast | chloroplast | peroxisome |  |
|  |  | 14888 | *Capsella grandiflora* | chloroplast | chloroplast | peroxisome |  |
|  |  | 15634 | *Tarenaya hassleriana* | - | chloroplast | chloroplast |  |
|  | APx-L | 3920 | *Arabidopsis thaliana* | chloroplast | chloroplast | chloroplast |  |
|  |  | 3921 | *Lycopersicon esculentum* | chloroplast | chloroplast | chloroplast |  |
|  |  | 5174 | *Vitis vinífera* | chloroplast | chloroplast | chloroplast |  |
|  |  | 6447 | *Medicago truncatula* | chloroplast | chloroplast | chloroplast |  |
|  |  | 7514 | *Arabidopsis lyrata* | chloroplast | chloroplast | chloroplast |  |
|  |  | 8024 | *Eucalyptus grandis* | chloroplast | chloroplast | chloroplast |  |
|  |  | 8211 | *Nicotiana tabacum* | chloroplast | chloroplast | chloroplast |  |
|  |  | 8227 | *Nicotiana tabacum* | - | - | golgi |  |
|  |  | 8435 | *Eucalyptus globulus* | chloroplast | chloroplast | chloroplast |  |
|  |  | 8470 | *Populus trichocarpa* | chloroplast | chloroplast | chloroplast |  |
|  |  | 8799 | *Cucumis sativus* | chloroplast | chloroplast | chloroplast |  |
|  |  | 8804 | *Citrus clementina* | chloroplast | chloroplast | chloroplast |  |
|  |  | 8940 | *Glycine max* | - | chloroplast | chloroplast |  |
|  |  | 8999 | *Glycine max* | chloroplast | chloroplast | chloroplast |  |
|  |  | 9051 | *Manihot esculenta* | chloroplast | chloroplast | chloroplast |  |
|  |  | 9167 | *Mimulus guttatus* | chloroplast | chloroplast | chloroplast |  |
|  |  | 9264 | *Prunus persica* | chloroplast | chloroplast | chloroplast |  |
|  |  | 9809 | *Thellungiella salsuginea* | chloroplast | chloroplast | chloroplast |  |
|  |  | 11383 | *Eucalyptus gunnii* | chloroplast | chloroplast | chloroplast |  |
|  |  | 12039 | *Linum usitatissimum* | chloroplast | chloroplast | mitochondria |  |
|  |  | 12041 | *Linum usitatissimum* | chloroplast | chloroplast | chloroplast |  |
|  |  | 14922 | *Capsella grandiflora* | chloroplast | chloroplast | chloroplast |  |
|  | hybrid | 15363 | *Lycopersicon esculentum* | - | - | cytosol |  |
|  |  | 1650 | *Mesembryanthemum crystallinum* | - | - | cytosol |  |
|  |  | 2230 | *Glycine max* | - | nucleus | golgi |  |
|  |  | 2469 | *Spinacia oleracea* | - | - | cytosol |  |
|  |  | 3446 | *Lactuca sativa* | chloroplast | chloroplast | chloroplast |  |
|  |  | 8227 | *Nicotiana tabacum* | - | - | golgi |  |
|  |  | 9327 | *Ricinus communis* | - | - | cytosol |  |
|  |  | 9331 | *Ricinus communis* | - | - | cytosol |  |
|  |  | 12221 | *Linum usitatissimum* | - | nucleus | nucleus |  |

| **Table S4**. APx-L protein-protein interactions deposited on plant.MAP database for *Arabidopsis thaliana* <http://plants.proteincomplexes.org/>) | | | | | | | | | |
| --- | --- | --- | --- | --- | --- | --- | --- | --- | --- |
| **CF-MS Score*** | **Gene Name 1** | **Gene Name 2** | **Orthogroup annotation 1** | | **Orthogroup annotation 2** | **Protein ID(s) 1** | **Protein ID(s) 2** | **TAIR**  **1** | **TAIR**  **2** |
| 0.4855 | APx-L, TL29 | PSBQ1,  PSBQ2 | | Ascorbate peroxidase-like, thylakoid lumenal 29 kDa protein | Oxygen-evolving enhancer protein | A0A1P8B8Y3 P82281 A0A1P8B8W6 | Q41932 Q9XFT3 | AT4G09010 | AT4G21280 AT4G05180 |
| 0.4158 | APx-L, TL29 | PSBP1,  PSBP2 | | Ascorbate peroxidase-like, thylakoid lumenal 29 kDa protein | Oxygen-evolving enhancer protein 2 | A0A1P8B8Y3 P82281 A0A1P8B8W6 | Q42029 O49344 | AT4G09010 | AT2G30790 AT1G06680 |
| 0.4119 | APx-L, TL29 | PRXQ | | Ascorbate peroxidase-like, thylakoid lumenal 29 kDa protein | Peroxiredoxin | A0A1P8B8Y3 P82281 A0A1P8B8W6 | Q9LU86 F4JBC9 A0A1I9LR27 | AT4G09010 | AT3G26060 |
| 0.3246 | APx-L, TL29 | NA | | Ascorbate peroxidase-like, thylakoid lumenal 29 kDa protein | Aldo/keto reductase family | A0A1P8B8Y3 P82281 A0A1P8B8W6 | Q9ZUX0 | AT4G09010 | AT2G27680 AT2G23660 |
| 0.323 | APx-L, TL29 | PPD6 | | Ascorbate peroxidase-like, thylakoid lumenal 29 kDa protein | PsbP domain-containing protein 6 | A0A1P8B8Y3 P82281 A0A1P8B8W6 | Q9LXX5 | AT4G09010 | AT3G56650 |
| 0.3113 | APx-L, TL29 | PPL1 | | Ascorbate peroxidase-like, thylakoid lumenal 29 kDa protein | PsbP-like protein | A0A1P8B8Y3 P82281 A0A1P8B8W6 | P82538 | AT4G09010 | AT3G55330 |
| 0.2954 | APx-L, TL29 | PPD4 | | Ascorbate peroxidase-like, thylakoid lumenal 29 kDa protein | Thylakoid lumenal 29.8 kDa protein | A0A1P8B8Y3 P82281 A0A1P8B8W6 | O49292 | AT4G09010 | AT1G77090 |
| 0.2612 | APx-L, TL29 | FKBP16-4 | | Ascorbate peroxidase-like, thylakoid lumenal 29 kDa protein | Peptidyl-prolyl cis-trans isomerase | A0A1P8B8Y3 P82281 A0A1P8B8W6 | Q9SR70 | AT4G09010 | AT3G10060 |
| 0.2481 | APx-L, TL29 | TL20.3 | | Ascorbate peroxidase-like, thylakoid lumenal 29 kDa protein | Thylakoid lumenal protein | A0A1P8B8Y3 P82281 A0A1P8B8W6 | Q8H1Q1 B6EUA5 A0A1P8AQ24 | AT4G09010 | AT1G12250 |
| 0.2464 | APx-L, TL29 | GDCST | | Ascorbate peroxidase-like, thylakoid lumenal 29 kDa protein | Glycine cleavage T-protein family | A0A1P8B8Y3 P82281 A0A1P8B8W6 | O65396 A0A2H1ZEA9 | AT4G09010 | AT1G11860 |
| 0.2461 | APx-L, TL29 | NA | | Ascorbate peroxidase-like, thylakoid lumenal 29 kDa protein | Thylakoid lumenal 19 kDa protein | A0A1P8B8Y3 P82281 A0A1P8B8W6 | P82658 | AT4G09010 | AT3G63540 |
| 0.2316 | APx-L, TL29 | PNSL5 | | Ascorbate peroxidase-like, thylakoid lumenal 29 kDa protein | Photosyntehtic NDH Subcomplex L 5 | A0A1P8B8Y3 P82281 A0A1P8B8W6 | Q9ASS6 | AT4G09010 | AT5G13120 |
| 0.2165 | APx-L, TL29 | DEGP1 | | Ascorbate peroxidase-like, thylakoid lumenal 29 kDa protein | Protease Do-like 1 | A0A1P8B8Y3 P82281 A0A1P8B8W6 | O22609 | AT4G09010 | AT3G27925 |
| 0.2115 | APx-L, TL29 | PSBO1,  PSBO2 | | Ascorbate peroxidase-like, thylakoid lumenal 29 kDa protein | Oxygen-evolving enhancer protein 1 | A0A1P8B8Y3 P82281 A0A1P8B8W6 | Q9S841 P23321 | AT4G09010 | AT3G50820 AT5G66570 |
| 0.1958 | APx-L, TL29 | ZCW7 | | Ascorbate peroxidase-like, thylakoid lumenal 29 kDa protein | NA | A0A1P8B8Y3 P82281 A0A1P8B8W6 | Q9SLT8 | AT4G09010 | AT1G59600 |
| 0.1906 | APx-L, TL29 | BGAL1 | | Ascorbate peroxidase-like, thylakoid lumenal 29 kDa protein | Beta-galactosidase | A0A1P8B8Y3 P82281 A0A1P8B8W6 | Q9SCW1 | AT4G09010 | AT3G13750 |
| 0.1819 | APx-L, TL29 | mMDH2 | | Ascorbate peroxidase-like, thylakoid lumenal 29 kDa protein | Malate dehydrogenase | A0A1P8B8Y3 P82281 A0A1P8B8W6 | Q9ZP06 Q9LKA3 A8MQK3 | AT4G09010 | AT3G15020 AT1G53240 |
| 0.1785 | APx-L, TL29 | PAHX | | Ascorbate peroxidase-like, thylakoid lumenal 29 kDa protein | Phytanoyl-CoA dioxygenase | A0A1P8B8Y3 P82281 A0A1P8B8W6 | Q9ZVF6 | AT4G09010 | AT2G01490 |
| 0.1774 | APx-L, TL29 | CYP26-2 | | Ascorbate peroxidase-like, thylakoid lumenal 29 kDa protein | Cyclophilin-like peptidyl-prolyl cis-trans isomerase family protein | A0A1P8B8Y3 P82281 A0A1P8B8W6 | F4HTT6 A0A1P8APN5 | AT4G09010 | AT1G74070 |
| *CF-MS scores > 0.5 correspond to ~90% true positive rate; scores > 0.2 correspond to ~50% true positive rate. | | | | | | | | | |
